# Effects of Isoxazolyl Steroids on Key Genes of Sonic Hedgehog Cascade Expression in Tumor Cells

**DOI:** 10.3390/molecules29174026

**Published:** 2024-08-26

**Authors:** Anna Aleksandrova, Arif Mekhtiev, Olga Timoshenko, Elena Kugaevskaya, Tatiana Gureeva, Alisa Gisina, Maria Zavialova, Kirill Scherbakov, Anton Rudovich, Vladimir Zhabinskii, Vladimir Khripach

**Affiliations:** 1 Institute of Biomedical Chemistry, 10 Building 8, Pogodinskaya Str., 119121 Moscow, Russia; anna142067@gmail.com (A.A.); ryzhakova.olga@list.ru (O.T.); elena.kugaevskaya@ibmc.msk.ru (E.K.); gureeva_t@mail.ru (T.G.); alisa.gisina@gmail.com (A.G.); mariag.zavyalova@gmail.com (M.Z.); kirill.soff@gmail.com (K.S.); 2 Institute of Bioorganic Chemistry, National Academy of Sciences of Belarus, Kuprevich Str., 5/2, 220084 Minsk, Belarus; rudovich.a.s@gmail.com (A.R.); vz@iboch.by (V.Z.); khripach@iboch.by (V.K.)

**Keywords:** steroids, isoxazoles, Hh receptors, cancer cell proliferation, MMP

## Abstract

Activation of the Hedgehog (Hh) signaling pathway is often associated with the progression of various types of cancer. The purpose of study was to search for inhibitors of the Hh signaling pathway among eight compounds belonging to the group of isoxazolyl steroids. The evaluation of the effectiveness of the compounds was based on the analysis of their cytotoxicity, effect on the cell cycle, on the expression of key Hh-signaling-pathway genes (Ptch1, Smo, and Gli1) and putative target genes MMP-2 and MMP-9. Four compounds with the most pronounced cytotoxic effect were identified: compounds **1**, **2** (HeLa cells) and **3**, **4** (A549 cells). Compounds **1** and **2** significantly reduced the expression of the Ptch1, Smo, Gli1 genes, but had the opposite effect on MMP-2 gene expression: Compound **1** increased it, and compound **2** decreased it. Compounds **3** and **4** did not have a noticeable inhibitory effect on the expression of the Shh pathway receptors, but significantly inhibited MMP-2 and MMP-9 expression. Thus, it was shown that inhibition of the Shh signaling pathway by isoxazolyl steroids can have the opposite effect on MMPs gene expression, which is what should be taken into account in further studies of these compounds as therapeutic agents.

## 1. Introduction

The Hedgehog pathway (Hh) is an evolutionarily conserved signal transduction from the cell membrane to the nucleus. It was originally discovered as a key pathway for embryonic development; in the adult, the Hh pathway is mostly inactive [1]. The main components of the mammalian Hh pathway include the following three Hh ligands: Shh (most active), Dhh and Ihh polypeptides; transmembrane receptor Patched1 (Ptch1); transmembrane receptor-like protein Smoothened (Smo) associated with the G-protein; and three transcription factors: Gli1, Gli2, and Gli3 [2]. Ptch1 contains a sterol-sensitive domain that is involved in the transport of sterols from the membrane; in the absence of Shh, this leads to inhibition of Smo activity [3]. Binding of the Shh ligand to Ptch1 blocks cholesterol transport, activates Smo, and triggers further intracellular signaling cascades involving Gli transcription factors (Gli1, Gli2, and Gli3) [4,5]. The direct targets of Gli are genes responsible for tissue patterning, cell proliferation, and regulators of cell survival, as well as genes of various components of the Hh pathway [6,7]. Activation of the Hh pathway and, in particular, Gli transcription factors is often associated with the progression of various types of cancer [2].

The relationship between the aberrant activation of Hh signaling and expression of matrix metalloproteinases (MMPs) has been shown [8]. MMPs are zinc-dependent enzymes that cleave extracellular matrix (ECM) components, initiate the processes of angiogenesis, invasion, and metastasis, and are involved in the regulation of cell proliferation, cell cycle, apoptosis, and adhesion [9]. MMPs are involved in all stages of tumor progression, affecting a variety of biological functions, including modification of signaling pathways, cytokine regulation, and tumor growth [10]. A significant role in these processes is played by MMP-2 and MMP-9, which are able to cleave type IV collagen. The latter is part of the basement membrane, which initiates the process of angiogenesis and gives tumor cells access to blood and lymphatic vessels [11]. The expression of MMP-2 and MMP-9 is increased in many types of cancer, including lung cancer, breast cancer and glioma, and cervical cancer and is considered an important prognostic factor [12,13,14,15,16].

Since the Hh pathway is involved in the development of many types of human malignant tumors, it is now considered as a potential therapeutic target. Steroid molecules are directly related to the mechanisms of regulation of Hh signaling activation. It can be assumed that synthetic steroids, similar in structure to cholesterol or cyclopamine, may be antagonists of the main receptors of the Hh signaling cascade, Smo and Gli. The aim of this study was to search for the Hh pathway inhibitors among compounds belonging to the group of steroid isoxazoles, which have shown a positive effect in the treatment of prostate cancer [17]. The evaluation of the effectiveness of the studied compounds was based on their cytotoxicity, effect on the cell cycle, as well as the expression of key genes of the Hh signaling pathway (Ptch1, Smo, Gli1) and putative target genes of molecules involved in tumor progression: MMP-2 and MMP-9. This study was carried out on cell lines of cervical cancer (HeLa) and lung adenocarcinoma (A549).

## 2. Results

### 2.1. Docking Results

Eight investigated compounds were docked to the binding site of cyclopamine of SMO protein. In total, 37 to 54 poses per compound were generated in ten runs. To evaluate the binding affinity of compounds and to determine the most possible binding mode, the average binding free energies for every cluster of every compound were calculated based on the values of the Autodock Vina scoring function. True binding modes were selected based on the average affinity of a cluster and the position of the ligand compared to the cyclopamine’s one. It is suggested that the studied compounds should share a similar binding mode with cyclopamine, e.g., C17-substituents would be oriented towards entry to the binding pocket, and the A-ring would be oriented to its bottom. So, we rejected clusters where C17-substituents were oriented toward the pocket’s bottom and selected only one cluster for each compound (Figure S1). The steroidal core of the investigated compounds bound in “upside down” conformation, e.g., it was flipped over compared to the core of cyclopamine, so the C19 atoms of cyclopamine and the studied compounds protruded in opposite directions (Figure S2).

The studied compounds share with cyclopamine the same hydrophobic interactions with residues L303, Y394, F484, and P513, assuming a similar profile of hydrophobic interactions (Table 1). Despite this, the investigated compounds totally lost the hydrogen bond with E518, which probably could be explained with the flipped-over conformation of the steroidal core. Instead of this, the 3-OH group of compounds **1**, **4**, **5**, and **7** formed H-bonds with D473, and the 3-OH group of compounds **4** and **7** formed additional H-bonds with R480. Also, all of the studied compounds formed additional H-bonds by its isoxazole moieties with Y207 and/or K395, depending on the system, except the compound **3**, which formed a H-bond by its pyridine fragment with K395.

**Figure 1:**
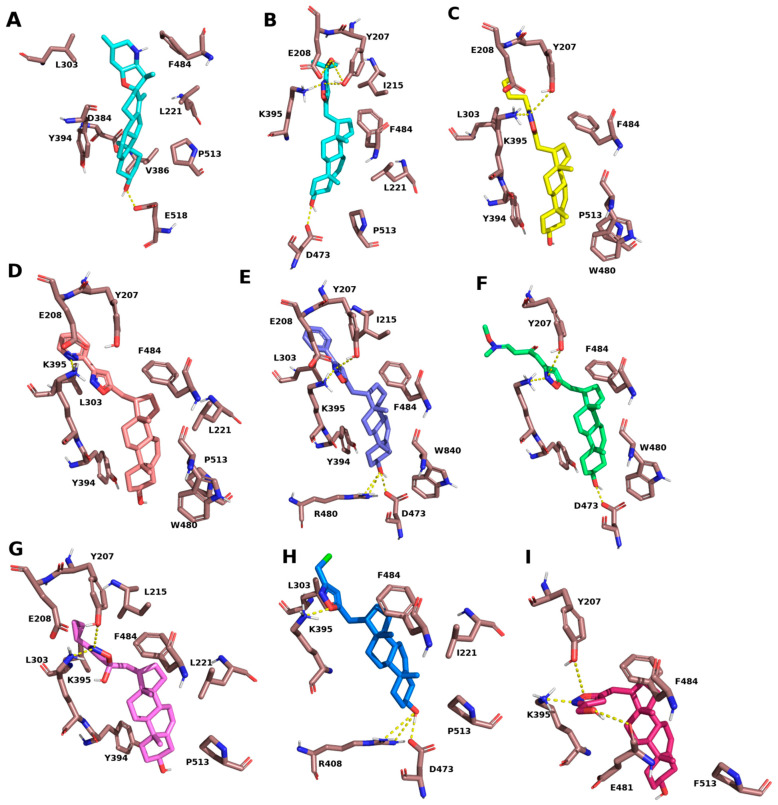
The binding modes of cyclopamine (**A**), **1** (**B**), **2** (**C**), **3** (**D**), **4** (**E**), **5** (**F**), **6** (**G**), **7** (**H**), and **8** (**I**). The yellow dotted lines are hydrogen bonds.

The average predicted affinities for each chosen cluster showed large negative values, lesser than −10 kcal/mol, which allows us to propose that the studied compounds are potent ligands of SMO, and the most potent ones are **3** and **4** (the predicted affinities are −12.1 kcal/mol for both compounds). The high predicted affinities and the additional H-bonds, formed by compounds, may indicate that they are more potent and specific inhibitors of the SMO receptor, compared to cyclopamine (Figure 1).

### 2.2. Cytotoxicity Analysis of Compounds ***1***–***8*** in HeLa Cells and A549 Cells

**Figure 2:**
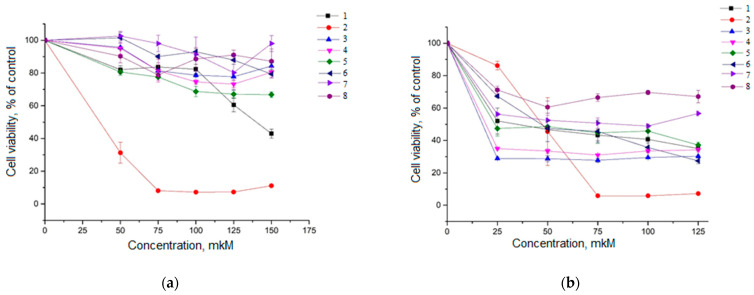
The effect of isoxazolyl steroids on the viability of HeLa cells (**a**) and A549 cells (**b**) during incubation for 48 h in DMEM medium with 10% FBS.

Cell lines HeLa and A549 were chosen as a model of human cancers expressing Hh receptors [18,19]. A study of isoxazolyl steroids as the proliferation regulators using the MTT assay showed that most of compounds possess moderate toxicity both in Hela and in A549 cells (Figure 2).

In HeLa cells, the most effective compounds were **1** (IC_50_ = 142 µM) and **2** (IC_50_ = 36 µM). They had the greatest toxicity and slowed down proliferation of (Figure 2a), while all other compounds did not exhibit toxic properties and not affected the growth of Hela cells. In A549 cells, the greatest cytotoxicity showed compounds **3** (IC_50_ = 16 µM) and **4** (IC_50_ = 19 µM), compared to other isoxazolyl steroids (Figure 2b). Thus, for each cell line, compounds with the greatest cytotoxicity were found.

### 2.3. Effect of Compounds ***1***, ***2***, ***3***, and ***4*** on the Cell Cycle

**Figure 3:**
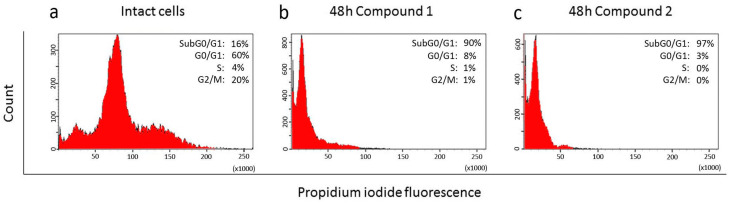
Flow cytometric analysis of DNA content in HeLa cells. (**a**) Intact HeLa cells; (**b**) HeLa cells incubated for 48 h with compound **1**; (**c**) HeLa cells incubated for 48 h with compound **2**. SubG0/G1 peaks on histograms indicate apoptotic cells; G0/G1, S, and G2/M—phases of cell cycle.

The cytostatic effect of compounds **1** and **2** on HeLa cells was evaluated based on cell cycle analysis. DNA content was analyzed by staining with propidium iodide (PI) with subsequent flow cytometry (Figure 3). An analysis of cell distribution in different cell cycle phases showed that treatment with both compounds caused a shift of almost all cells to the left in the region of the subG0/G1 peak. The percentage of apoptotic cells was 90% and 97% for **1** and **2**, respectively. This indicates a strong cytotoxic effect from both compounds.

**Figure 4:**
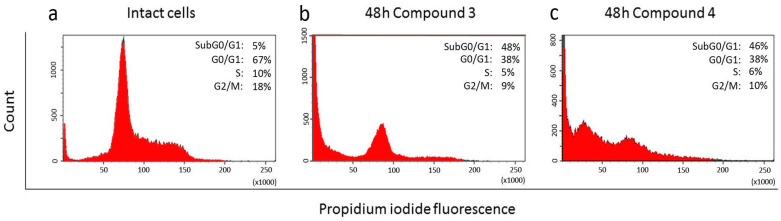
Flow cytometric analysis of DNA content in HeLa cells. (**a**) Intact A549 cells; (**b**) A549 cells incubated for 48 h with compound **3**; (**c**) A549 cells incubated for 48 h with compound **4**. SubG0/G1 peaks on histograms indicate apoptotic cells; G0/G1, S, and G2/M—phases of cell cycle.

The cytotoxic effects of compounds **3** and **4** in A549 cells were visualized using propidium iodide (PI) flow cytometry (Figure 4). A small difference in their effects on A549 cell proliferation was observed. Both compounds significantly increased the number of events at the subG0/G1 peak compared to the untreated cells. The percentage of apoptotic cells was 46% and 48% for **3** and **4**, respectively. These data indicate a moderate cytotoxic effect from both compounds.

### 2.4. Effect of Compounds ***1*** and ***2*** on the Expression of Hh Signaling Pathway Genes in HeLa Cells

**Figure 5:**
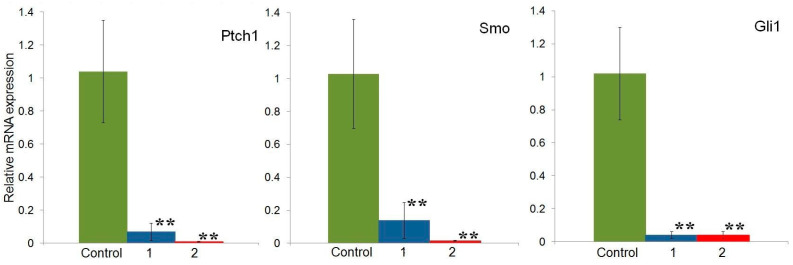
Effect of isoxazolyl steroids **1** and **2** on expression of Hh pathway receptors genes in HeLa cell line. ** *p* < 0.05.

To study the effect of compounds **1** and **2** on the expression of the Ptch1, Smo, and Gli1 genes, HeLa cells were treated with isoxazolyl steroids at concentrations corresponding to the IC_50_ for each compound: 146 μM for **1** and 37 μM for **2** (Figure 2a). As can be seen in Figure 5, both compounds caused a significant suppression of Ptch1, Smo, and Gli1 mRNA expression compared to untreated cells.

### 2.5. Effect of Compounds ***1*** and ***2*** on the Expression of MMP-2 Gene in HeLa Cells

**Figure 6:**
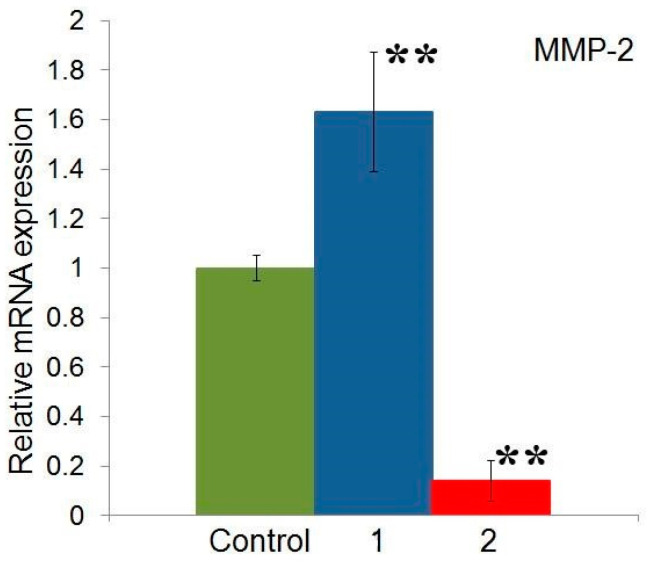
Effect of isoxazolyl steroids **1** and **2** on expression of MMP-2 and MMP-9 genes in HeLa cell line. ** *p* < 0.05.

A study of the gene expression of MMP-2 and MMP-9 in HeLa cells showed that there was no expression of MMP-9, which does not contradict the data of other authors, in whose studies the gene expression of MMP-9 in HeLa cells was either absent [14,20] or was determined at a very insignificant level [21]. As for the gene expression of MMP-2, its level significantly increased under the influence of compound **1** and, on the contrary, significantly decreased under the influence of compound **2** (Figure 6).

### 2.6. Effect of Compounds ***3*** and ***4*** on the Expression of Hh Signaling Pathway Genes in A549 Cells

**Figure 7:**
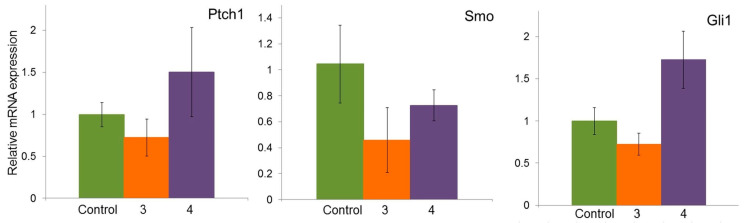
Effect of isoxazolyl steroids **3** and **4** on expression of Hh pathway receptors genes in A549 cell line.

To study the effect of compounds **3** and **4** on the expression of Ptch1, Smo, and Gli1 genes, cell line A549 was treated in concentrations corresponding to the IC_50_ for each compound: 16 µM for **3** and 19 µM for **4** (Figure 2b). Treatment of cells with compound **3** tended to decrease the mRNA expression of Ptch1, Smo, and Gli1 genes compared to untreated cells (Figure 7). When cells were treated with compound **4**, the gene expression levels of Ptch1 and Gli1 increased, while Smo gene expression decreased. It should be noted that all observed changes in the expression of the Ptch1, Smo, and Gli1 genes were not statistically significant.

### 2.7. Effect of Compounds ***3*** and ***4*** on the Gene Expression of MMP-2 and MMP-9 in A549 Cells

**Figure 8:**
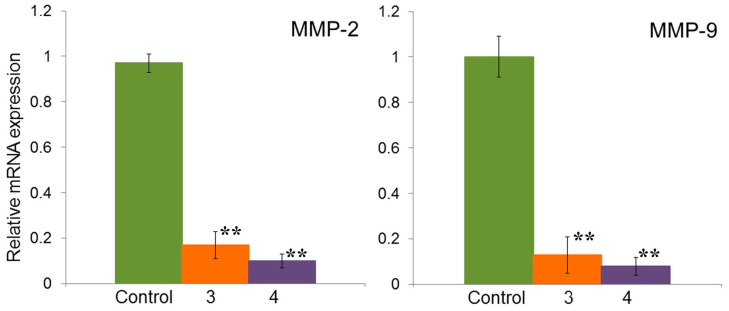
Effect of isoxazolyl steroids **3** and **4** on expression of MMP-2 and MMP-9 genes in A549 cells line. ** *p* < 0.05.

In contrast to the HeLa cell line, which lacked MMP-9 expression, A549 cells showed expression of both MMP-2 and MMP-9. Compounds **3** and **4** significantly suppressed the gene expression of MMP-2 and MMP-9 (Figure 8), despite the fact that they did not show themselves as potential inhibitors of the Hh signaling pathway in this cell line (Figure 7).

### 2.8. Effect of Compounds ***1*** and ***2*** on Proliferation in the MCF-7 Cell Line

The MCF-7 cell line is estrogen-sensitive; in its cells, the content of estrogen receptors (ER) is maintained at a constant level with exponential cell growth [22] and used for in vitro studies of cytotoxicity of antitumor pharmaceuticals. The cytotoxicity in the MCF-7 cells was studied by the MTT test method, which indicates the antiproliferative effect of cells that bore estrogen receptors.

## 3. Discussion

Recent data show that the Hh signaling pathway is involved at different stages of tumor progression in various types of cancer. Dysregulation of any of the components of the Hh pathway leads to its aberrant activation and ultimately to malignant transformation [2]. It has been shown that in gastric and prostate cancer, activation of Hh signaling is associated with the processes of invasion and metastasis [23]. In esophageal and pancreatic cancer, its activation is observed both in the early stages of tumor development and in metastatic tumors [24,25]. Hh signaling is involved in the development of lung cancer [18]. Its activation has been shown to promote epithelial–mesenchymal transition in lung squamous cell carcinoma [26]. Hh signaling is involved in the development of cervical cancer; the expression of all signaling molecules of this pathway (Shh, PTCH, Smo, Gli-1, Gli-2, and Gli-3) is increased in cervical carcinoma and is very rarely found in normal cervical epithelium [27,28]. Due to the fact that steroid molecules are directly related to the mechanisms regulating the activation of Hh signaling, an assumption was made that synthetic steroids, similar in structure to cholesterol or cyclopamine, may be antagonists of the main receptors of the Hh signaling cascade: Smo and Gli. For research, we used eight compounds (**1**–**8**) belonging to the group of steroid derivatives of isoxazoles: effective inhibitors of testosterone biosynthesis, which have shown a positive effect in the treatment of prostate cancer [17]. Studying their effect on Hh signaling as well as on the expression of MMP-2 and MMP-9, which are involved in tumor progression, is interesting from the point of view of creating drugs with antitumor activity that can be used in various types of human cancer.

At the first stage we carried out a comparative analysis of the binding affinity of all compounds to the Smo receptor using the docking method. From the eight investigated molecules, isoxazolyl steroids **3** and **4** showed the highest predicted affinity to the SMO receptor. The high predicted binding free energies and their binding mode, highly similar to cyclopamine’s one, allow us to assume that compounds **3** and **4** are possible antagonists of the SMO receptor. It corresponds to the obtained experimental data, which shows that isoxazolyl steroid **3** can partially suppress SMO-dependent signaling in the A549 cell line. Despite this, according to the docking data, these compounds should have the same effects on the SMO receptor, as they have highly similar binding modes. It can be assumed that the observed difference in H-bonding patterns for these compounds is an artifact of docking. The small translation and rotation of compound **3** will allow it to take a position similar to **4** and form an identical H-bonding pattern.

Our analysis of the cytotoxic effects of the studied isoxazolyl steroids allowed us to select the leading compounds for each cell line: in the HeLa cell line, **1** and **2** showed the greatest cytotoxicity, and in the A549 cell line, **3** and **4** showed the greatest cytotoxicity (Figure 2).

A study of the genes of the Hh signaling cascade showed that in the HeLa cell line, compounds **1** and **2** reliably suppressed the transcription of the Ptch, Smo, and Gli1 genes (Figure 5). This apparently indicates their ability to influence the canonical Hh signaling pathway, upon activation of which signal transmission from the cell membrane to the nucleus occurs with the participation of the Shh, Ptch, and Smo proteins, which ultimately leads to the translocation of Gli transcription factors into nuclei and activation of their target genes [19].

In the A549 cell line, compounds **3** and **4** did not appear to be potential inhibitors of the Hh pathway (Figure 7), although in the presence of compound **3,** there was some tendency to suppress the expression of the Ptch, Smo, and Gli genes. In the presence of compound **4**, the gene expression of Smo decreased and the expression of the Ptch and Gli genes increased. However, the data obtained for both compounds were not statistically significant.

Taking into account the tendency to increase gene expression of Gli1 with a decrease in gene expression of Smo in the presence of compound **4**, it can be assumed that Gli expression in this case was stimulated by factors related to the non-canonical Hh signaling pathway. It has been shown that non-canonical activation of Hh signaling can occur both due to post-translational modifications of Gli1 and cross-talk with other oncogenic signaling pathways that contribute to the transcriptional activation of Gli1 [29]. MMPs are involved at all stages of tumor progression, promoting tumor growth and development, including modification of signaling pathways, regulation of cytokines and tumor growth [10]. MMP-2 and MMP-9 destroy type IV collagen, which is part of the basement membrane, STM, and ensure the invasion of tumor cells into surrounding tissues [11]. MMP expression is regulated by many factors, including extracellular matrix proteins, growth factors and cytokines, as well as hormonal receptors [30]. The expression of MMP-2 and MMP-9 is increased in many types of cancer, including lung cancer, breast cancer and glioma, and cervical cancer and is considered an important prognostic factor [12,13,14,15,16].

A study of the effect of compounds **1** and **2** on the gene expression of MMP-2 in the HeLa cell line showed that their effect is opposite: against the background of compound **2**, the expression of MMP-2 decreases, while against the background of compound **1**, on the contrary, an increase in MMP-2 gene expression is observed (Figure 6).

It has been suggested that the observed difference in MMP-2 expression is due to the structural features of these compounds, which can be considered as ligands of steroid receptors. It was previously shown that compound **1** is an antagonist of androgen receptors (AR) with moderate activity, in contrast to compound **2**, which did not exhibit the properties of either an antagonist or an AR agonist [17]. In recent years, ARs have been found to be closely associated with the occurrence and progression of cancers of the female reproductive system; loss of AR expression is commonly observed in cervical cancer [31]. It is also known that in this pathology the expression of estrogen receptors (ER) is reduced [32]. Since compounds **1** and **2** belong to the group of steroid compounds, an assumption was made that they may also interact with estrogen receptors.

**Figure 9:**
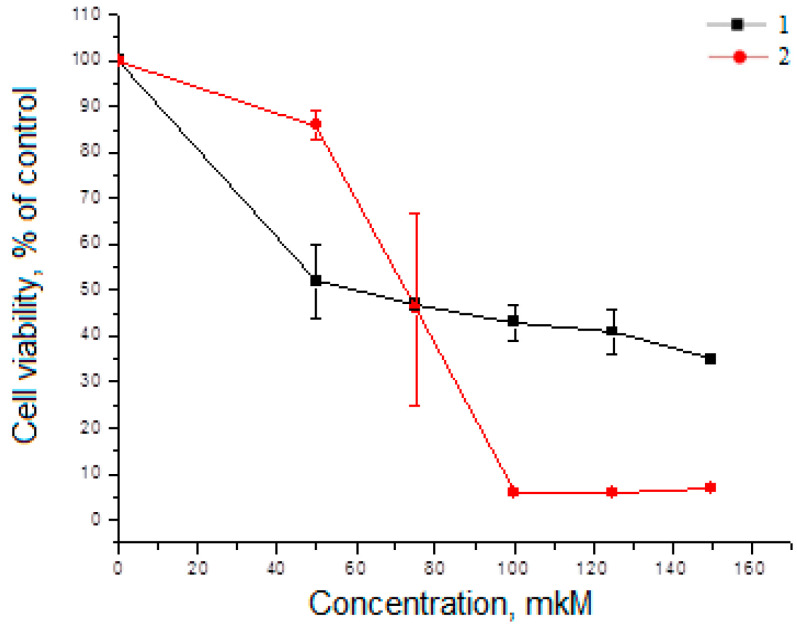
The effect of isoxazolyl steroids **1** and **2** on the viability of MCF-7 cells during incubation for 48 h in DMEM medium with 10% FBS.

To test hypothesis, has been compared the effects of compounds **1** and **2** on the viability of the estrogen-sensitive cell line MCF-7, which is rich in ER and is involved in the regulation of the proliferation of these cells [22,33]. It has been shown that compound **2** inhibits the proliferation of MCF-7 cells, in contrast to compound **1**, which has no effect on this process (Figure 9). It can be assumed that compounds **1** and **2** have directly opposite effects on the AR and ER: being an AR antagonist, compound 1 does not interact with the ER, while compound **2** does not interact with the AR but is an ER antagonist. An increase in the level of MMP-2 expression in HeLa cells against the background of compound **1** is associated with the manifestation of antagonism to AR, and the ability of compound **2** to reduce the level of gene expression of MMP-2 may be due not only to gene transcription blockage of the Ptch, Smo, and Gli1 receptors but also to interaction with ER. Thus, the multitarget effect of compound **2** observed in HeLa cells makes its further study promising as an antitumor agent for cervical cancer.

As for estrogen receptors, it seems unlikely that any of the studied compounds could be their ligands, despite the high activity of **2** in MCF-7 cells. The binding site of ERα is adopted to bind the planar A-ring of estrogens with no C19 atom, but the A-ring of compound **2** has non-planar ‘chair’ conformation. But it is possible that the A-ring of **2** is converted to an aromatic ring by aromatase, which may allow these compounds to bind to the ER.

A study of the expression of the MMP-2 and MMP-9 genes in the A549 cell line against the background of compounds **3** and **4** showed that both compounds significantly reduced their expression level (Figure 8). Taking into account that both compounds did not exhibit significant inhibitory activity on the expression of the Hh signaling pathway genes, it can be assumed that the effect of compounds **3** and **4** on the expression of MMP-2 and MMP-9 in A-549 cells was mediated by their interaction with other signaling pathways and molecules; in the A-549 cell line, gene expression of MMP-2 and MMP-9 depended on the activation of the FAK-ERK signaling pathway [34], and in non-small cell lung cancer, it was stimulated by interleukin IL-17A/IL-17RA through the p38 MAPK signaling pathway [35].

## 4. Materials and Methods

### 4.1. Materials

Isoxazolyl steroids **1**–**8** (Table 2) were prepared by us earlier [17]. In the article above, detailed methods for their synthesis are described and all the spectroscopic data to evaluate the purity of the obtained compounds are provided.

### 4.2. Molecular Docking

The structure of the SMO receptor in a complex with cyclopamine (PDB ID: 4o9r) was obtained from Protein DataBank. The obtained complex was used as a target for molecular docking. The docking was performed on the binding pocket of cyclopamine. All non-protein molecules were removed from the complex. The structures of compounds **1**–**8** were built in the SYBYL package. Further, ligand and protein molecules were energy-minimized in SYBYL 8.1 by Tripos forcefield using Powell’s method. Partial atomic charges for both protein and ligand were calculated with the Gasteiger–Hückel method. All docking calculations were performed with AutoDock Vina [36] in 10 repetitions for each ligand. The obtained poses were clustered with a self-written python script, based on the GROMOS algorithm [37]. The RMSD cutoff of 1.25 Å was used to discriminate between clusters. Binding poses were selected based on the average binding energies of cluster members, calculated by Autodock Vina scoring function and on their spatial positions. Protein–ligand complexes obtained from docking were additionally energy-minimized. An analysis of binding features for the chosen poses was performed with PLIP server [38].

### 4.3. Cells and Culture

Commercially available standard human cell cultures obtained from the American Tissue and Cell Collection (ATCC, Manassas, VA, USA) were used for this work: HeLa cervical carcinoma, A549 lung carcinoma, and MCF-7 breast adenocarcinoma cells. 

Cell lines were cultivated in 25 cm^2^ Corning flasks in 4 mL DMEM medium (PanEco, Moscow, Russia) with 10% FBS and antibiotics, penicillin at a concentration of 50 units/mL, and streptomycin at 50 μg/mL (PanEco, Russia). The cells were transplanted 2–3 times a week. For detachment, the monolayer was treated with a solution of Versene (PanEco, Russia), then with a solution of trypsin (Gibco, Waltham, MA, USA). Trypsinolysis was inhibited by DMEM culture medium with 10% serum content. Cell counts were performed using Vi-CellTMXR (Beckman Coulter, Brea, CA, USA). For cell counting, a single cell suspension was prepared in a ratio of 1:10 (100 µL of cell suspension, 900 µL of medium).

### 4.4. Cytotoxicity Evaluation (MTT Test)

The cell lines were cultivated in 96-well plates at 37 °C in atmosphere of 5% CO_2_ in a DMEM medium with the addition of L-glutamine (2 mM), antibiotics (50 units per mL of penicillin and 50 μg/mL of streptomycin), and 10% of FBS. The cells were incubated in a serum medium with the tested compounds at various concentrations for 48 h. PBS (10 μL) containing MTT (5 mg/mL) was added to each well, and the cells were incubated at 37 °C for 4 h. The culture medium was removed, DMSO (100 μL) was added to each well, a plate was vortexed for 20 min, and then the optical absorbance in each well was measured at 570 nm in a Multiskan Spectrum Microplate Reader (Thermo Scientific, Waltham, MA, USA). The MTT test readings were averaged for three independent determinations. Readings of MTT test in the absence of the tested compounds were taken as 100%. 

### 4.5. Cell Cycle Analysis

The cells were suspended in DMEM/F12 culture medium supplemented with 10% FBS and placed in 6-well plates at a concentration of 10^6^ cells/well. After cell adherence, the tested compounds were added into the wells at a concentration of IC_50_. Forty-eight hours post-treatment, the cells were washed in PBS and then harvested using Versene solution and 0.25% trypsin. Then, the cells were centrifuged at 250× *g* in PBS for 3 min twice. Washed cell pellets from each well were resuspended in a small amount of PBS and fixed with 0.5 mL 70% ethanol for 30 min. The fixed cells were incubated with 200 μL of RNase A (200 μg/mL) solution in PBS supplemented with 0.1% *v*/*v* Triton X-100 for 30 min in the dark at RT, at 37 °C for 30 min, and finally stained with 100 μL of 1 mg/mL propidium iodide (PI) at 20 °C for 10 min. The fluorescence of the stained cells was measured using the flow cytometer BD FACSAria III. The obtained data were analyzed using BD FACS Diva software version 7 (BD Biosciences, Franklin Lakes, NJ, USA).

### 4.6. Real-Time PCR

Total RNA was extracted using the <<SV Total RNA Isolation System>> (Promega, Madison, WI, USA), and reverse transcription was carried out using the <<MMLV RT kit>> (Evrogen, Moscow, Russia) according to the manufacturer’s instructions. cDNA was synthesized with hexaprimes; the final concentration of RNA was 50 ng/mL. The samples were stored at −20 °C.

Real-time PCR was performed with qPCRmix-HS SYBR (Evrogen, Russia) on a CFX96 Real-Time system (BioRad, Hercules, CA, USA). Three replicates were performed for each biological sample. The expression levels of Ptch1, Smo, Gli1,MMP-2, MMP-9, TIMP-1, and TIMP2 of each replicate were normalized against glyceraldehyde phosphate dehydrogenase (GAPDH) cDNA using the 2^−ΔΔCT^ method [39].

Specific primers were selected using Gene Bank Nucleotide Sequence Database. The structure of the primer was evaluated by means of the Oligo 4.1 Primer Analysis Software.

The PCR primer sequences are as follows:

Ptch1—Forward: 5′ tgtgcgctgtcttccttctg 3′, Ptch1—Reverse: 5′ acggcactgagcttgattc 3′;

Smo—Forward: 5′ cccatccctgactgtgagat 3′, Smo—Reverse: 5′ caggtacgcctccagatgag 3′;

Gli1—Forward: 5′ caggtatgtaaccccctgga 3′, Gli1—Reverse: 5′ tcccccaatttttctggaag 3′;

MMP-2—Forward: 5′ gagttggcagtgcaatacct 3′, MMP-2—Reverse: 5′ gccatccttctcaaagttgt 3′;

MMP-9—Forward: 5′ gatcattcctcagtgccgga 3′, MMP-9—Reverse: 5′ ttcagggcgaggaccataga 3′;

TIMP-1—Forward: 5′ ggggcttcaccaagacctac 3′, TIMP-1—Reverse: 5′ ggaagcccttttcagagcct 3′;

TIMP-2—Forward: 5′ ggtctcgctggacgttggag 3′, TIMP-2—Reverse: 5′ ggagccgtcacttctcttg 3′;

GAPDH—Forward: 5′ accacagtccatgccatcac 3′, GAPDH—Reverse: 5′ tccaccaccctgttgctgta 3′.

### 4.7. Statistical Analysis

The experimental values were expressed as mean SEM. The differences between the control and treated groups were evaluated using Student’s *t* test and analysis of variance (ANOVA). *p* < 0.05 was taken as significant.

## 5. Conclusions

The effectiveness of compounds from the group of steroid derivatives of isoxazoles on the expression of genes of the Hh signaling pathway (Ptch1, Smo, and Gli1) and matrix proteinases MMP-2 and MMP-9 was assessed on the HeLa and A549 cell lines. It was shown that some of the studied compounds, along with inhibition of the expression of key genes of the Hh signaling pathway, also reduced the expression of MMP-2 and MMP-9, which should have a general inhibitory effect on the development of the tumor process. It is worth noting that compound **1**, on the contrary, caused an increase in the expression of MMP-2 against the background of inhibition of Hh signaling, which may aggravate the process of tumor progression. Thus, the results indicate that the effect of steroidal isoxazole derivatives on MMP expression can serve as a criterion for selecting compounds for further studies as therapeutic agents.

## Figures and Tables

**Table 1:** Key interactions of studied compounds with binding pocket of SMO receptor.

Compound	Hydrophobic Interactions	H-Bonds		Average Affinity for Cluster (kcal/mol)
		Residue	Length, Å	
Cyclopamine	F221, L303, L384, V386, Y394, F484, P513	E518	2.7	-
**1**	I215, L221, F484, P513	Y207	3.7	
		E208	3.0	
		E208	3.4	
		K395	2.9	−10.9
		D473	2.7	
**2**	Y207, E208, L303, Y394, W480, F484, P513	Y207	3.0	
		K395	2.6	−10.7
**3**	Y207, E208, L221, L303, Y394, W480, F484, P513	K395	2.8	−12.1
**4**	Y207, E208, I215, L303, Y394, W480, F484	Y207	3.7	
		K395	2.7	
		R480	3.6	
		R480	3.1	−12.1
		D473	3.1	
**5**	Y207, Y394, W480, F484	Y207	4.0	−10.1
		K395	2.9	
		D473	2.6	
		D473	4.1	
**6**	Y204, E208, I215, L221, L303, Y394, F484, P513	Y207	3.7	−11.1
		K395	3.6	
**7**	L221, L303, F484, P513	K384	2.5	−10.6
		R480	3.9	
		R480	3.8	
		D473	2.7	
**8**	F484, P513	Y207	3.8	−10.5
		K395	3.2	
		E481	3.5	

**Table 2:** Structures of the investigated isoxazolyl steroids.

No	Name	Structure
**1**	17β-((3-(2-hydroxypropan-2-yl)isoxazol-5-yl)methyl)androst-5-en-3β-ol	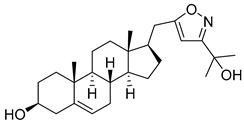
**2**	17β-((3-butylisoxazol-5-yl)methyl)androst-5-en-3β-ol	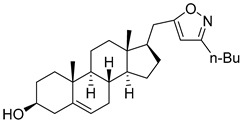
**3**	3β-hydroxy-17β-((3-(pyridin-2-yl)isoxazol-5-yl)methyl)androst-5-ene	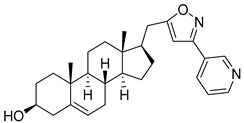
**4**	(17β)-17-((3-phenylisoxazol-5-yl)methyl)androst-5-en-3β-ol	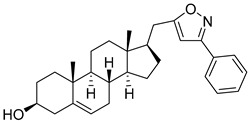
**5**	(*E*)-1-((17*R*)-3β-hydroxyandrost-5-en-17-yl)-4-(methoxy(methyl)amino)but-3-en-2-one	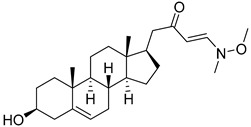
**6**	3-cyclopropyl-5-(((17*R*)-3β-hydroxyandrost-5-en-17-yl)methyl)-4,5-dihydroisoxazol-5-ol	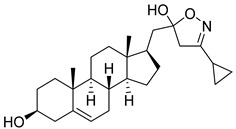
**7**	(17*R*)-17-((3-(chloromethyl)isoxazol-5-yl)methyl)androst-5-en-3β-ol	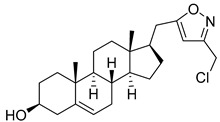
**8**	17β-((3-(hydroxymethyl)isoxazol-5-yl)methyl)androst-5-en-3β-ol	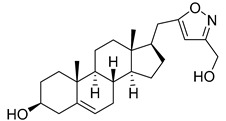

## Data Availability

Data are contained within this article and Supplementary Materials.

## Supplementary Materials

The following supporting information can be downloaded at https://www.mdpi.com/article/10.3390/molecules29174026/s1, Figure S1: Selected clusters of the docked compounds; Figure S2: The supposed “upside down” binding mode of the studied compounds.

## Abbreviations

AR	androgen receptor
DMEM	Dulbecco’s Modified Eagle Medium
DMSO	dimethylsulfoxide
ECM	extracellular matrix
ER	estrogen receptor
FBS	fetal bovine serum
Hh	Hedgehog pathway
MMPs	metalloproteinases
PBS	phosphate-buffered saline
PI	propidium iodide
RMSD	root-mean-square deviation
Smo	The protein that carries the Hh signal across the membrane is the oncoprotein and G-protein coupled receptor (GPCR) Smoothened (Smo)
