# Supplementary material for: Effects of Isoxazolyl Steroids on Key Genes of Sonic Hedgehog Cascade Expression in Tumor Cells
Source: Molecules. 2024 Aug 26;29(17):4026. doi: 10.3390/molecules29174026 (PMC11396458; doi:10.3390/molecules29174026)
Supplement: Supplementary file 1 [file molecules-29-04026-s001.zip › molecules-3112033-supplementary.pdf]

Supporting information to:

Anna Aleksandrova Arif Mekhtiev, Olga Timoshenko, Elena Kugaevskaya, Tatiana Gureeva, Alisa Gisina, Maria Zavialova, Kirill Scherbakov, Anton Rudovich, Vladimir Zhabinskii and Vladimir Khripach

Effects of Isoxazolyl Steroids on Key Genes of Sonic Hedgehog Cascade Expression in Tumor Cells

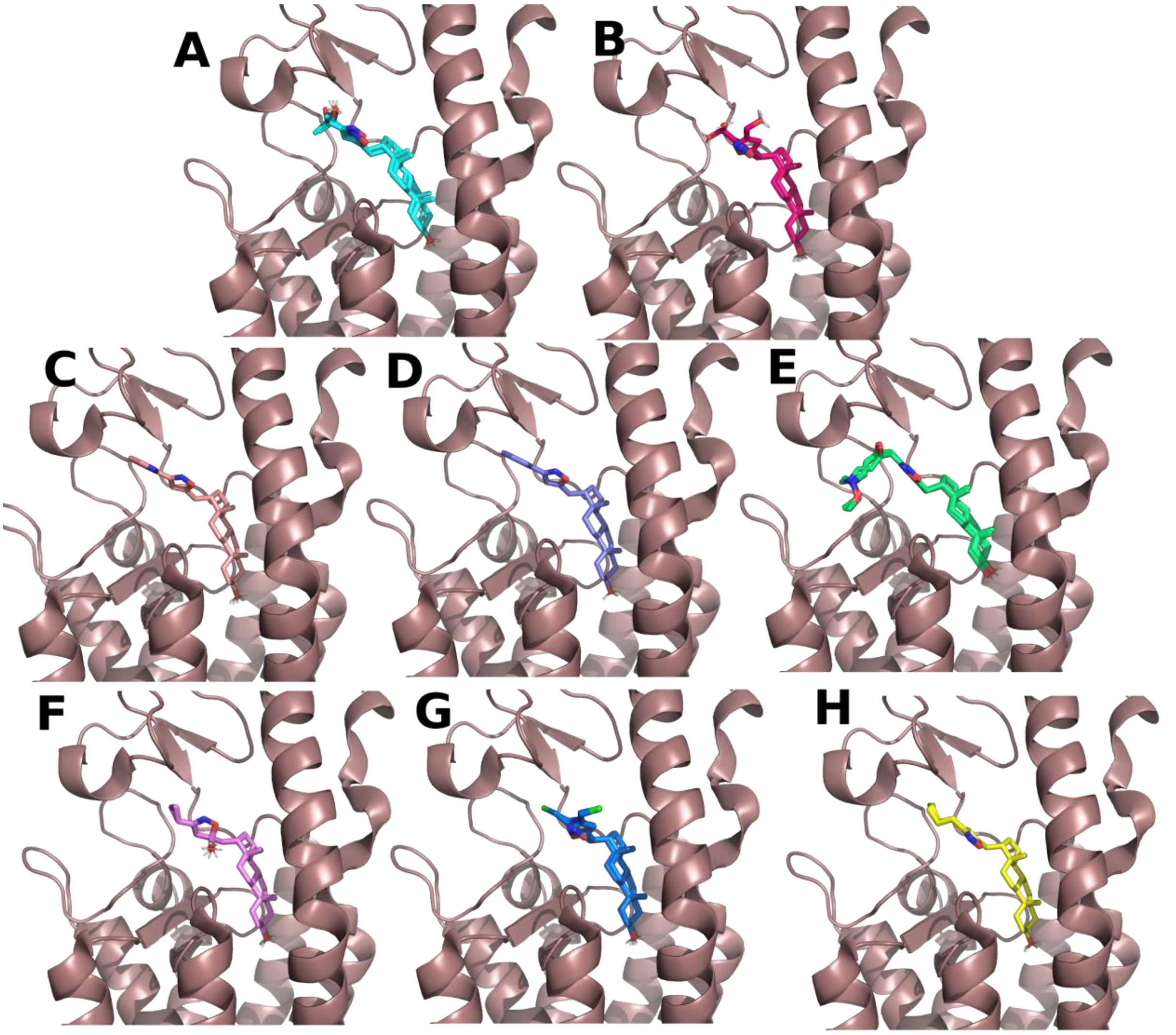

Figure S1. Selected clusters of the docked compounds. Each cluster contained from 5 to 10 binding poses. In every cluster the positions of steroidal cores are identical, the main differences in binding modes are observed in conformations of the C17-substituent of the D-ring.

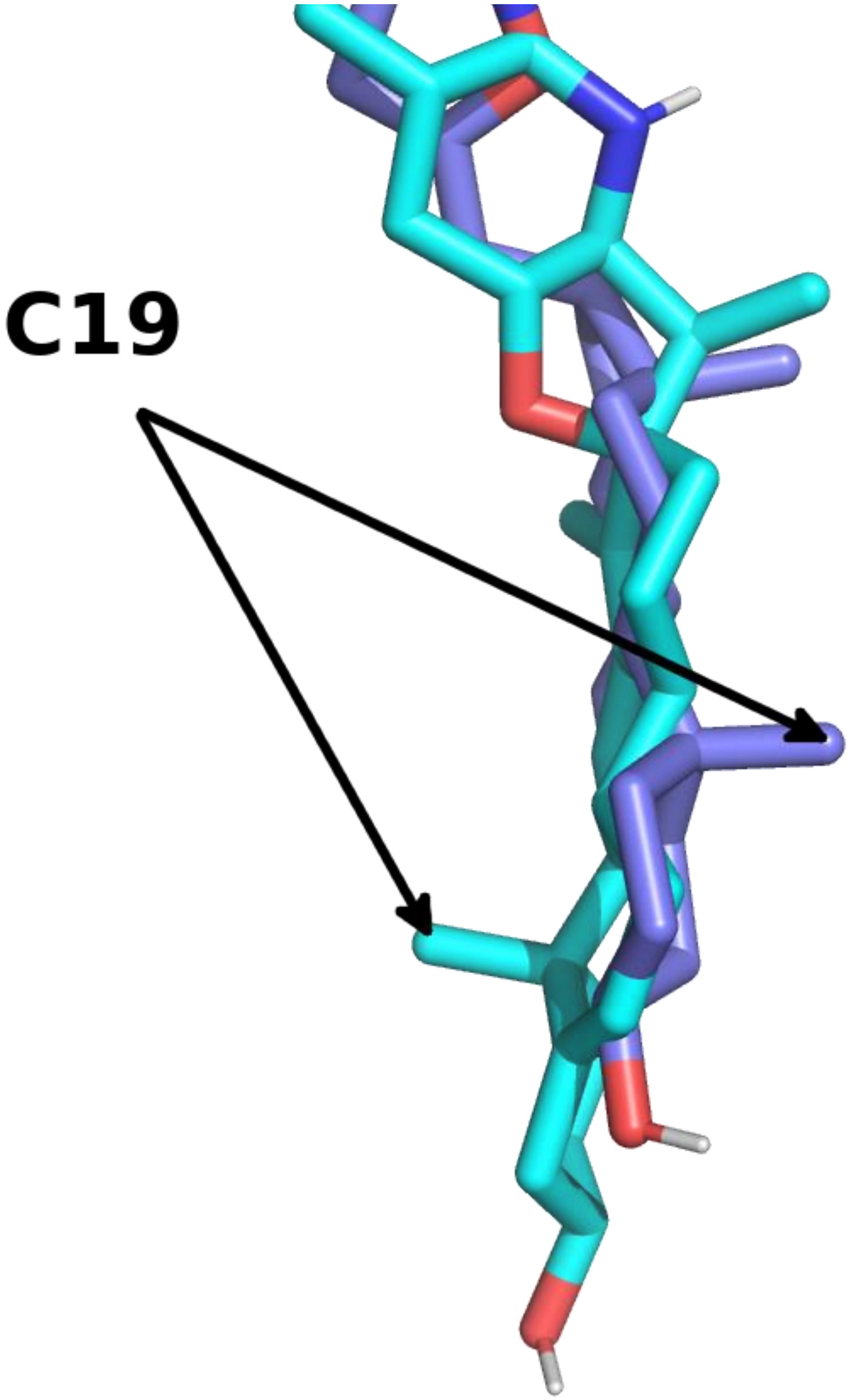

Figure S2. The supposed "upside down" binding mode of the studied compounds. The steroidal cores of compounds **1 - 8** are flipped over compared to cyclopamine`s one. The C19 atoms of the cyclopamine and studied compounds are oriented in opposite directions.
